# Social inequalities and untreated dental caries in 5-year-old children: an intersectional analysis based on SB Brasil 2023

**DOI:** 10.1590/1980-549720260014.supl.1

**Published:** 2026-06-29

**Authors:** Monarko Nunes de Azevedo, João Victor Alves Alencar, Mary Anne de Souza Alves França, Marcos Vinicius Rodrigues de Melo, Matheus Ribeiro dos Santos, Gustavo Adolfo Martins Mendes, Edsaura Maria Pereira

**Affiliations:** I Universidade Federal de Goiás - Goiânia (GO), Brazil.; II Universidade Federal do Sul da Bahia - Porto Seguro (BA), Brazil.

**Keywords:** Dental caries, Social determinants of health, Health inequalities, Intersectionality

## Abstract

**Objective:**

To identify intersectional vulnerability profiles among 5-year-old children with untreated dental caries and to analyze associated social inequalities in Brazil.

**Methods:**

This is a population-based, cross-sectional study using data from the National Survey of Oral Health (SB Brasil 2023). Five-year-old children examined in the survey were included. The variables used to identify profiles were: race/skin color, household income, maternal level of education, beneficiaries of social welfare programs, and region of residence. For profile comparison, the variables “last dental appointment” and “presence of piped water at home” were used. Descriptive, bivariate (χ²), Two-Step Cluster, and binary and multinomial logistic regression analyses were performed.

**Results:**

A total of 7,185 children were analyzed, of whom 44.8% presented untreated dental caries. Higher prevalence values were observed among children with low maternal education (51.9%), Black (49.9%) and Indigenous (75.0%), low income (54.1%), living in the North (56.3%) region, and beneficiaries of social welfare programs (53.0%) (p<0.001). Three profiles were identified: Profile A (boys, Black, from the Northeast, low income, and high dependence on social welfare programs) showed lower probability of dental appointment in the past 2 years (OR 0.59; 95%CI 0.48-0.73; p<0.001); Profile B (boys, White, from the Northeast, low income, and beneficiaries of social welfare programs) showed worse conditions regarding the presence of piped water at home (OR 0.38; 95%CI 0.19-0.76; p=0.006); and Profile C (girls, Black, from the North, higher income, and lower dependence on social welfare programs) concentrated the best conditions for dental appointment and piped water (p<0.001).

**Conclusion:**

Untreated dental caries among 5-year-old children reflects structural inequities combining racial, socioeconomic, territorial, and income-related inequalities.

## INTRODUCTION

Dental caries is one of the most prevalent chronic diseases in the world, generating relevant clinical, functional, and psychosocial impacts throughout life^1^. Despite being susceptible to prevention and treatment, its distribution is marked by important social, economic, and territorial inequalities, reflecting patterns of health inequities observed in both high and low-income countries^2^ ^,^ ^3^ ^,^ ^4^ ^,^ ^5^.

In 2019, approximately 514 million children had untreated dental caries in deciduous teeth worldwide, corresponding to an overall average prevalence of 43%, with values above 40% in more than 2/3 of the countries^3^. In the Brazilian context, dental caries persists as a priority harm in childhood, particularly at 5 years of age, the age group internationally used as a reference for epidemiological monitoring of early tooth decay and its consequences^6^ ^,^ ^7^.

There is evidence that the occurrence and persistence of dental caries are strongly associated with social determinants of health, which operate through gradients related to income, level of education, race/skin color, living conditions, access to drinking water, and availability of health services^2^ ^,^ ^4^ ^,^ ^8^ ^,^ ^9^. These determinants do not operate individually; on the contrary, they tend to overlap and interact with each other, producing more complex patterns of vulnerability as social markers - such as race/skin color, social class, and territory - accumulate^3^ ^,^ ^5^ ^,^ ^8^.

In this sense, the perspective of intersectionality, originally introduced by Crenshaw and later expanded into the field of collective health^8^, has emerged as a relevant reference to understand how inequality axes articulate themselves in the production of inequalities. In the field of oral health, authors of recent studies have advocated the incorporation of intersectionality into the study of inequalities, highlighting that explanations based on isolated determinants underestimate the complexity of the social processes involved^8^ ^,^ ^10^.

However, the application of intersectionality in the epidemiology of oral health is still limited. Most researchers adopt methodological approaches that deal with determinants as isolated variables, making it difficult to capture combined patterns of social disadvantage^7^. Within this context, clustering techniques, such as Two-Step Cluster, have been recommended as a methodological alternative to identify vulnerability profiles, allowing to analyze how social markers combine in the production of inequalities^3^. Although useful, these strategies remain underused in the dental literature, especially in childhood.

The Brazilian reality offers a relevant context for such analyses. The country has profound racial, territorial, and socioeconomic inequalities historically structured, which have repercussions on the use, opportunity, and problem-solving capacity of oral health care^6^ ^,^ ^9^. Authors have recently suggested that Black, Indigenous, poor children or those living in underdeveloped regions face accumulated barriers to dental care, even in the context of the Brazilian Unified Health System (SUS)^4^.

In addition, according to national data, the prevalence of 5-year-old children with at least one deciduous tooth with untreated dental caries decreased from 53.4% in 2010 to 41.2% in 2023, showing a reduction in the period, but with persistence of important social and territorial inequalities^5^.

In this context, the National Survey of Oral Health (SB Brasil 2023) represents an opportunity to investigate inequalities in oral health in childhood, given its national scope, standardized methodology, and availability of social, clinical, and care variables^5^. Nonetheless, intersectional analyses based on this database have not yet been explored.

Considering the identified gaps, in the present study, we aim to identify intersectional profiles of vulnerability in 5-year-old children with untreated dental caries and to analyze their social inequalities.

## METHODS

### Study design

This is a cross-sectional, population-based, and analytical study based on data from SB Brasil 2023.

### Study participants

The study population was composed of children of both sexes, aged 5 years, examined in the survey (national sample, representative by Federation Unit [FU], region, capital/small cities); records with absence of data on dental caries experience, inconsistencies in the forms, or lack of information in the essential demographic, socioeconomic, and territorial variables were excluded.

### Variables

The decayed, extracted, and filled teeth (deft) index, which quantifies decayed, missing, and filled deciduous teeth, was used as an indicator of dental caries experience. Children with deft=0 were classified as “without tooth decay.” For the “≥1 decayed tooth” classification, only the “decayed” component of the index was used, considering the presence of at least one deciduous tooth with active caries (d>0).

For the identification of intersectional profiles, only records of children with untreated dental caries were considered (decayed component >0). Social and demographic variables were selected based on the literature on social determination of health and intersectionality, including the categorical variables race/skin color (white, Black^11^ [Black and mixed-race], Asian, and Indigenous); monthly household income (less than 1 minimum wage, from 1 to 2 minimum wages, and more than 2 minimum wages); maternal level of education (illiterate/Youth and Adult Education [*Educação de Jovens e Adultos* - EJA], incomplete or complete Elementary School, incomplete or complete High School, incomplete or complete Higher Education); region of residence (North, Northeast, Midwest, Southeast, and South); and beneficiary of social welfare programs (yes or no), including the *Bolsa Família* Program and the Continuous Cash Benefit (cash transfer programs of the Brazilian government).

After creating the profiles, comparative analyses were carried out between the identified groups using variables related to living conditions and access to services, considering the last dental appointment (has never visited a dentist, in the last 2 years, and over 2 years ago), employed as a proxy for access to care, and the presence of piped water at home (with plumbing or without plumbing), used as a proxy of household infrastructure.

The categories followed the standardization of SB Brasil 2023 and were kept in the original form or clustered according to epidemiological relevance^12^ ^,^ ^13^.

### Data source

The used data derive from SB Brasil 2023. Crude data from SB Brasil 2023 were obtained from the General Coordination of Oral Health of the Brazilian Ministry of Health, upon formal request. This database gathers demographic, socioeconomic, territorial, clinical and dental services information collected in a standardized way throughout the country.

To evaluate the quality of the data and reduction of potential biases, checks of completeness and logical consistency of the information were carried out. Records referring to children without information on the deft index or with inconsistencies in the answers were excluded prior to the statistical analysis.

### Study sample

All records available for the period were considered, ensuring the representativeness of the sample. This approach avoided random sampling, as the data were secondary and covered a significant universe of the target population.

### Statistical methods

In the descriptive analysis, absolute and relative frequencies of the demographic, socioeconomic, territorial, and clinical-care characteristics of the total sample were calculated. The bivariate associations between the presence of dental caries and social markers were tested in the total sample by Pearson’s χ² test, adopting a 5% significance level.

To identify intersectional vulnerability profiles, a Two-Step Cluster analysis was performed using the previously specified social and demographic variables. The log-likelihood distance measure was used, and the Bayesian Information Criterion (BIC) guided the selection of the optimal number of clusters. The resulting profiles were described according to their predominant characteristics.

Subsequently, differences between the profiles were investigated regarding living conditions and access to care. These analyses were exclusively conducted among the individuals classified in the profiles, considering that the latent variable “profile” is not attributable to incomplete cases and cannot be imputed without distorting the probabilistic structure of the clusters.

Binary logistic regression was used for the variable “piped water at home” (reference: without plumbing) and multinomial logistic regression was considered for the variable “dental appointment” (reference: has never visited a dentist). The reference categories were chosen because they represent situations of greater vulnerability and are epidemiologically relevant as a comparative baseline.

Odds ratios (OR) and 95% confidence intervals (95%CI) were estimated. The models were estimated in an unadjusted way, as the profiles result from the simultaneous combination of the social markers previously incorporated into the cluster analysis, and it is not necessary to include these same markers as additional covariates in the subsequent models.

All statistical analyses were performed using the IBM SPSS Statistics software, version 23.0 (IBM Corp., Armonk, NY, United States), and were conducted without sampling weights, as it is an internal exploration of social profiles and not population estimates.

The study followed the STROBE (Strengthening the Reporting of Observational Studies in Epidemiology)^14^ recommendations, according to which transparency and completeness are recommended in the presentation of observational studies.

## Data Availability Statement

The entire dataset that supports the results of this study is available upon request to the corresponding author. The dataset is not publicly available due to the integration of databases carried out by the authors.

## RESULTS

Of the total of 40,720 individuals registered in SB Brasil 2023, 7,198 were children aged 5 years, of which 7,185 (17.6%) were included in the analysis after the exclusion of 13 records without information on the deft index.

In the demographic, socioeconomic, and territorial profile (Table 1), we observed a balanced distribution between sexes (51.1% boys; 48.9% girls) and predominance of residents in the Northeast (36.6%) and North (24.6%) regions. Regarding race/skin color, 61.9% were classified as Black (Black and mixed-race) and 34.6% as white. As for maternal level of education, 40.7% had complete or incomplete High School and 15.5% had Higher Education degree. In addition, 48.7% of families were beneficiaries of social welfare programs. From the clinical-care point of view, 40.1% of the children had never visited a dentist, and 44.8% had untreated dental caries.

**Table 1.** Demographic, socioeconomic, territorial, and clinical-care characteristics of 5-year-old children. Brazil, 2023 (n=7,185).

Characteristics	n (%)
Sex	
Boys	3,670 (51.1)
Girls	3,515 (48.9)
Region	
North	1,767 (24.6)
Northeast	2,633 (36.6)
Southeast	842 (11.7)
South	859 (12.0)
Midwest	1,084 (15.1)
Race/skin color	
White	2,488 (34.6)
Black	4,450 (61.9)
Asian	76 (1.1)
Indigenous	24 (0.3)
Not informed	147 (2.0)
Maternal level of education	
Illiterate/Youth and Adult Education	658 (9.2)
Incomplete or complete Elementary School	1,847 (25.7)
Incomplete or complete High School	2,926 (40.7)
Incomplete or complete Higher Education	1,115 (15.5)
Not informed	639 (8.9)
Income (minimum wage)^a^	
Less than 1	1,978 (27.5)
From 1 to 2	1,661 (23.1)
More than 2	1,158 (16.1)
Not informed	2,388 (33.2)
Family beneficiary of social welfare program	
Yes	3,499 (48.7)
No	3,029 (42.2)
Not informed	657 (9.1)
Last dental appointment	
Has never visited a dentist	2,878 (40.1)
In the last 2 years	3,658 (50.9)
Over 2 years ago	334 (4.6)
Not informed	315 (4.4)
Piped water at home	
With plumbing	6,570 (91.4)
Without plumbing	171 (2.4)
Not informed	444 (6.2)
Tooth decay	
No decayed teeth	3,965 (55.2)
≥1 decayed tooth	3,220 (44.8)

*The household income variable was self-reported in ranges of minimum wages. During the collection period of SB Brasil 2023 (March/2022 to March/2024), the Brazilian minimum wage ranged from BRL 1,212.00 (2022), BRL 1,302.00 (Jan.-Apr./2023), to BRL 1,320.00 (May/2023-2024).

In the bivariate analysis (Table 2), we verified a significant association between untreated dental caries and sex, region, race/skin color, maternal level of education, household income, and beneficiaries of social welfare programs and presence of piped water at home (p<0.05).

**Table 2.** Association between demographic, socioeconomic, territorial characteristics and presence of untreated dental caries in 5-year-old children. Brazil, 2023 (n=7,185).

Characteristics	No decayed teeth n (%)	≥1 decayed tooth n (%)	p-value
Sex			
Boys	1,982 (54.0)	1,688 (46)	0.040
Girls	1,983 (56.4)	1,532 (43.6)	
Region			
North	773 (43.7)	994 (56.3)	<0.001
Northeast	1,416 (53.8)	1,217 (46.2)	
Southeast	576 (68.4)	266 (31.6)	
South	563 (65.5)	296 (34.5)	
Midwest	637 (58.8)	447 (41.2)	
Race/skin color			
White	1,607 (64.6)	881 (35.4)	<0.001
Black	2,229 (50.1)	2,221 (49.9)	
Asian	43 (56.6)	33 (43.4)	
Indigenous	6 (25.0)	18 (75.0)	
Maternal level of education			
Illiterate/Youth and Adult Education	354 (53.8)	304 (46.2)	<0.001
Incomplete or complete Elementary School	888 (48.1)	959 (51.9)	
Incomplete or complete High School	1,586 (54.2)	1,340 (45.8)	
Incomplete or complete Higher Education	804 (72.1)	311 (27.9)	
Income (minimum wage)			
Less than 1	908 (45.9)	1,070 (54.1)	<0.001
From 1 to 2	875 (52.7)	786 (47.3)	
More than 2	818 (70.6)	340 (29.4)	
Family beneficiary of social welfare program			
Yes	1,644 (47.0)	1,855 (53.0)	<0.001
No	1,938 (64.0)	1,091 (36.0)	
Last dental appointment			
Has never visited a dentist	1,605 (55.8)	1,273 (44.2)	0.715
In the last 2 years	2,003 (54.8)	1,655 (45.2)	
Over 2 years ago	185 (55.4)	149 (44.6)	
Piped water at home			
With plumbing	3,610 (54.9)	2,960 (45.1)	<0.001
Without plumbing	69 (40.4)	102 (59.6)	

The prevalence of dental caries was higher among boys (46%) than among girls (43.6%); among residents of the North (56.3%) and Northeast (46.2%) regions, compared to the Southeast (31.6%) and South (34.5%); among Indigenous (75%) and Black (49.9%) children, compared to white (35.4%). The lowest prevalence occurred among children of mothers with Higher Education degree (27.9%), while the highest prevalence occurred among children of mothers with Elementary Education (51.9%). The prevalence was also higher in families with household income less than 1 minimum wage (54.1%), in beneficiaries of social welfare programs (53%), and in households without piped water (59.6%) (p<0.001). The variable “last dental appointment” did not present a statistically significant association with dental caries (p=0.715) (Table 2).

In the Two-Step Cluster analysis (Table 3), variables with greater weight for defining the profiles were beneficiary of social welfare programs, race/skin color, and household income, while region, maternal level of education, and sex had lower influence. We identified three intersectional profiles: Profile A, composed mainly of boys (55.7%), from the Northeast (49.4%), Black (100%), children of mothers with incomplete or complete High School (45.3%), with household income less than 1 minimum wage (66%), and beneficiaries of social welfare programs (95.9%); Profile B, composed mainly of boys (50.2%), from the Northeast (41.5%), white (92%), children of mothers with incomplete or complete High School (52.3%), with household income less than 1 minimum wage (63.1%), and beneficiaries of social welfare programs (90.8%); and Profile C, composed mainly of girls (51.2%), from the North (31.4%), Black (64.4%), daughters of mothers with incomplete or complete High School (45%), with household income greater than 2 minimum wages (43.8%), and who were not beneficiaries of social welfare programs (91.3%).

**Table 3.** Frequency of demographic, socioeconomic, and territorial characteristics, according to the profiles of 5-year-old children with untreated dental caries. Brazil, 2023 (n=3,220).

Characteristics	Profile A 50.6% (1,030)	Profile B 16.0% (325)	Profile C 33.5% (682)
Sex			
Boys	55.7	50.2	48.8
Girls	44.3	49.8	51.2
Region			
North	33.5	22.8	31.4
Northeast	49.4	41.5	20.4
Southeast	3.6	4.9	11.6
South	2.7	17.5	17.9
Midwest	10.8	13.2	18.8
Race/skin color			
White	0.0	92.0	34.8
Black	100	0.0	64.4
Asian	0.0	4.9	0.3
Indigenous	0.0	3.1	0.6
Maternal level of education			
Illiterate/Youth and Adult Education	8.4	8.0	11.1
Incomplete or complete Elementary School	41.7	35.7	21.7
Incomplete or complete High School	45.3	52.3	45.0
Incomplete or complete Higher Education	4.5	4.0	22.1
Income (minimum wage)			
Less than 1	66.0	63.1	14.8
From 1 to 2	34.0	30.8	41.3
More than 2	0.0	6.2	43.8
Family beneficiary of social welfare program			
Yes	95.9	90.8	8.7
No	4.1	9.2	91.3

In the comparison between profiles and indicators of access and living conditions (Table 4), children of Profile C presented a higher proportion of recent dental visits, while Profiles A and B concentrated the lower frequencies of use of services. Concerning household infrastructure, Profile B presented a higher proportion of households without piped water.

**Table 4.** Comparison of the profiles of 5-year-old children with untreated dental caries, according to last dental appointment and presence of piped water at home. Brazil, 2023 (n=3,220).

Profile	Last dental appointment				Piped water at home		
	Has never visited a dentist (%)	In the last 2 years (%)	Over 2 years ago (%)	p-value^§^	With plumbing (%)	Without plumbing (%)	p-value^§^
Profile A*	47.7	48.0	4.4	<0.001	96.8	3.2	0.018
Profile B^†^	43.2	51.2	5.6		94.4	5.6	
Profile C^‡^	35.0	59.2	5.8		97.8	2.2	

*Boys, from the Northeast, Black, mother with incomplete or complete High School, household income less than one minimum wage, and beneficiary of social welfare program; ^†^boys, from the Northeast, white, mother with incomplete or complete High School, household income less than one minimum wage, and beneficiary of social welfare program; ^‡^girls, from the North, Black, mother with incomplete or complete High School, household income greater than two minimum wages, and no beneficiary of social welfare program; ^§^Pearson’s χ^2^.

According to logistic regression analyses (Table 5), Profiles A and B presented lower odds of access to dental services and piped water compared to Profile C. The difference was more pronounced in Profile A for dental access and Profile B for access to piped water.

**Table 5.** Odds ratio and 95% confidence intervals of last dental appointment and presence of piped water at home, according to the profiles of 5-year-old children with untreated dental caries. Brazil, 2023 (n=3,220).

Profile	Category	OR	95%CI	p-value^§^
Piped water at home				
Profile A*	Without plumbing	0.68	0.37-1.26	0.217
Profile B^†^	Without plumbing	0.38	0.19-0.76	0.006
Profile C^‡^	Ref.	-	-	-
Last dental appointment				
Profile A*	Over 2 years ago	0.55	0.35-0.87	0.011
Profile B^†^	Over 2 years ago	0.78	0.43-1.42	0.415
Profile C^‡^	Ref.	-	-	-
Profile A*	In the last 2 years	0.59	0.48-0.73	<0.001
Profile B^†^	In the last 2 years	0.70	0.53-0.93	0.012
Profile C^‡^	Ref.	-	-	-

OR: Odds ratio; CI: Confidence interval.

*Boys, from the Northeast, Black, mother with incomplete or complete High School, household income less than one minimum wage, and beneficiary of social welfare program; ^†^boys, from the Northeast, white, mother with incomplete or complete High School, household income less than one minimum wage, and beneficiary of social welfare program; ^‡^girls, from the North, Black, mother with incomplete or complete High School, household income greater than two minimum wages, and no beneficiary of social welfare program; ^§^for last dental appointment, multinomial logistic regression (reference category: has never visited a dentist). For piped water at home, binary logistic regression (reference category: without plumbing).

## DISCUSSION

In this study, we verified a high prevalence of untreated dental caries in 5-year-old children in Brazil, with higher frequencies among Black and Indigenous children, from the North and Northeast regions, with low income, and beneficiaries of social welfare programs. As per the intersectional analysis, we identified three distinct vulnerability profiles, with Profile A being the most vulnerable and Profile C, the least vulnerable. We observed a lower probability of dental appointment and worse access to piped water among the most vulnerable profiles.

The unequal distribution of untreated dental caries in the country indicates that this disease reflects structural patterns of inequality, as already pointed out by authors of national and international investigations that associate the disease with conditions of social vulnerability^4^ ^,^ ^10^ ^,^ ^15^ ^,^ ^16^ ^,^ ^17^. Considering the high prevalence observed, especially in the North and Northeast regions and among Black and Indigenous children, failure to tackle the disease reflects the persistence of structural inequalities in the supply and access to dental care^18^ ^,^ ^19^ ^,^ ^20^. These results suggest that untreated dental caries, more than a clinical issue, is a concrete expression of social determination of health^21^ ^,^ ^22^ ^,^ ^23^ ^,^ ^24^.

The intersectional analysis of the data, operationalized by the identification of three vulnerability profiles, reinforces this understanding. Profile A, predominantly composed of Black children, of low income, low maternal education, mostly residents in the Northeast, and with high dependence on social welfare programs, represents the point of great social vulnerability^25^ ^,^ ^26^. The overlapping of racial, economic, and territorial markers constitutes greater vulnerability to the nontreatment of dental caries, which is compatible with studies whose authors point to structural barriers in access and continuity of care for historically marginalized populations^6^ ^,^ ^9^ ^,^ ^18^ ^,^ ^19^ ^,^ ^20^.

Profile B, mainly composed of white children, presented socioeconomic vulnerability and greater absence of piped water, indicating that poverty constitutes structural barriers to oral health^27^. However, it differs from Profile A, in which economic vulnerability is added to racial and territorial markers, thus increasing the inequalities^8^ ^,^ ^10^. In the Brazilian context, there is evidence that poverty is not experienced in a homogeneous way between racial groups, reflecting intersectional effects of skin color/race and class^28^.

Profile C, although with better economic and access conditions, is not exempt from the marks of racial inequality, which demonstrates that race remains a structuring axis of care opportunities, as discussed in studies whose authors apply the concept of intersectionality to oral health^10^ ^,^ ^25^ ^,^ ^29^ ^,^ ^30^.

Untreated dental caries results, therefore, from the accumulation of social disadvantages. The direct association with low income, low maternal education, and dependence on social welfare programs corroborates that dental treatment, even in the context of SUS, is not carried out in a universal or equitable way^27^ ^,^ ^31^ ^,^ ^32^. Families in poverty have less conditions to access preventive and rehabilitation services in addition to facing logistical, cultural, and institutional barriers^33^ ^,^ ^34^.

Low family education limits the ability to understand the importance of early care, while economic vulnerability restricts time and means to seek care^35^ ^,^ ^36^. Although the maternal level of education was associated with untreated dental caries in the present study, it is worth noting the relatively homogeneous distribution of this variable in the sample, with predominance of complete or incomplete High School and presence of a portion with Higher Education. This composition decreases the educational contrast between subgroups, which may have reduced their discriminatory power in the creation of profiles. This finding is consistent with changes in recent decades related to the expansion of women’s education in Brazil, in which higher formal levels of education can coexist with economic and racial vulnerability^37^ ^,^ ^38^.

The relationship between household infrastructure and oral health is worthy of attention. The absence of piped water was associated with higher prevalence of untreated dental caries, indicating that material living conditions are part of the set of factors that shape the possibility of adequate oral hygiene and use of inputs such as fluoride toothpaste^5^ ^,^ ^16^. In this sense, the observed disease cannot be interpreted only in clinical terms, but as an expression of structural inequalities in access to basic rights, particularly relevant in early childhood^21^ ^,^ ^26^.

Another relevant finding was the absence of an association between dental appointment and not presenting untreated dental caries. This result suggests that oral health care in childhood does not depend exclusively on occasional contact with the dental service, but on factors that involve continuity of care, organization of the care network, health education, family support, and living conditions. The appointment alone does not ensure the problem-solving capacity of the care, which is compatible with analyses whose authors point to structural and organizational limitations in primary care, suppressed demands, and practices centered on emergency and mutilating procedures^18^ ^,^ ^19^ ^,^ ^27^ ^,^ ^28^ ^,^ ^31^ ^,^ ^39^.

Untreated dental caries can be understood, therefore, as an expression of inequities in care, and not just as prevention failure. While initial access to services may have expanded, the problem-solving and continuity capacity is still uneven^29^. The nontreatment of the disease thus represents the final stage of exclusion: getting sick and not being treated. This phenomenon evidences a qualitative inequality, a “problem-solving capacity inequity,” in which vulnerable groups have less chance to complete treatment and achieve rehabilitation.

The fact that the variables race/skin color, maternal level of education, income, and social welfare program have greater weight in the formation of untreated dental caries profiles than clinical or care factors reinforces the understanding of social determination of oral health^5^ ^,^ ^22^ ^,^ ^35^ ^,^ ^36^. Intersectionality contributes to understanding that these processes do not act alone - the effect of poverty is aggravated by racism, and both are intensified by territorial contexts of exclusion. This approach breaks with traditional perspectives that isolate variables, showing that social determinants interact with each other^30^ ^,^ ^32^.

Childhood is a period when inequalities can manifest early. The presence of untreated dental caries at the age of 5 represents not only an immediate clinical problem, but an indicator of early accumulation of vulnerabilities that may affect school development, self-esteem, nutrition, and general well-being^24^ ^,^ ^40^. The persistence of these inequalities since early childhood points to a process of generational injustice, in which adverse social conditions in childhood shape more unequal life trajectories^35^.

This study presents limitations inherent in the use of secondary data from SB Brasil 2023^5^, such as dependence on standardized information and lack of broader contextual variables, for example, coverage of oral health teams, primary care characteristics, and local environmental indicators^21^ ^,^ ^35^. In addition, the variable “last dental appointment” does not allow to distinguish the type of care (preventive, curative, or emergency), which can underestimate qualitative inequalities in care^18^.

Another relevant aspect refers to the structure of the analyzed variables, which have different levels (family, household, and territorial) and were modeled on the same individual level, which can generate loss of contextual information. Furthermore, the cross-sectional nature of the study makes it impossible to establish causal relationships between social determinants and the experience of untreated dental caries, and findings should be interpreted with caution.

As a strength, we mention the use of a national database, standardized methodology, and application of intersectional analysis by the Two-Step Cluster analysis, which empirically operationalized social and racial dimensions in the field of oral health^25^ ^,^ ^29^ ^,^ ^30^. This strategy allowed us to capture the intertwining of social and racial dimensions, offering a more deep and contextualized view of iniquities. Future studies may advance by incorporating contextual variables, such as the density of oral health teams, coverage of the Family Health Strategy (FHS), and indicators of urban infrastructure, to understand broader levels of social determination^21^ ^,^ ^27^.

The present findings have direct implications for public policies. Coping with untreated dental caries requires strategies that combine clinical actions with structural interventions^36^ ^,^ ^41^. It is necessary to strengthen primary care in oral health, increase water fluoridation, develop anti-racist and intersectional educational actions, and ensure longitudinal follow-up of the most vulnerable children^22^ ^,^ ^23^ ^,^ ^42^ ^,^ ^43^. Policies must explicitly recognize the social markers of inequality, incorporating racial, territorial, and social equity as criteria for prioritization.

Untreated dental caries is, therefore, a mirror of Brazilian inequalities, being concentrated among Black, poor children from the North and Northeast regions, which points to an unequal distribution of the right to health and childhood^4^ ^,^ ^16^ ^,^ ^26^ ^,^ ^32^. According to the profile analysis, the intersection of social markers - such as race/skin color, income, maternal education, and territory - is not additive, but produces specific arrangements of vulnerability, reaffirming that the disease expresses the social determination of health. Moreover, to combat the disease, there must be public policies on racial, territorial and social equity and a SUS capable of reducing inequalities since the first years of life.
