# Supplementary material for: Social inequalities and untreated dental caries in 5-year-old children: an intersectional analysis based on SB Brasil 2023
Source: Rev Bras Epidemiol. 2026 Jun 29;29(Suppl 1):e260014supl1. doi: 10.1590/1980-549720260014.supl.1 (PMC13325560; doi:10.1590/1980-549720260014.supl.1)
Supplement: Translation

# Translation: Cárie dentária não tratada em crianças de 5 anos e desigualdades sociais: uma análise interseccional com base no Levantamento Nacional de Saúde Bucal 2023

**DOI:** 10.1590/1980-549720260014.supl.1.2

**Authors:** Monarko Nunes de Azevedo, João Victor Alves Alencar, Mary Anne de Souza Alves França, Marcos Vinicius Rodrigues de Melo, Matheus Ribeiro dos Santos, Gustavo Adolfo Martins Mendes, Edsaura Maria Pereira

**Affiliations:** I Universidade Federal de Goiás - Goiânia (GO), Brasil.; II Universidade Federal do Sul da Bahia - Porto Seguro (BA), Brasil.

**Keywords:** Cárie dentária, Determinantes sociais da saúde, Desigualdades de saúde, Interseccionalidade

## Abstract

**Objetivo:**

Identificar perfis interseccionais de vulnerabilidade em crianças de 5 anos com cárie dentária não tratada e analisar desigualdades sociais associadas no Brasil.

**Métodos:**

Estudo transversal de base populacional com dados do Levantamento Nacional de Saúde Bucal (SB Brasil) 2023. Foram incluídas crianças de 5 anos examinadas no inquérito. As variáveis utilizadas para identificação dos perfis foram: raça/cor, renda familiar, escolaridade da mãe, participação em programas sociais e região de residência. Para comparação entre perfis foram: última consulta com dentista e presença de água canalizada no domicílio. Realizaram-se análises descritivas, bivariadas (qui-quadrado), análise *Two-Step Cluster* e regressão logística binária e multinomial.

**Resultados:**

Foram analisadas 7.185 crianças, das quais 44,8% apresentaram cárie dentária não tratada. As maiores prevalências ocorreram entre crianças com baixa escolaridade da mãe (51,9%), negras (49,9%) e indígenas (75%), de baixa renda (54,1%), do Norte (56,3%) e beneficiárias de programas sociais (53%) (p<0,001). Identificaram-se três perfis: Perfil A (meninos, negros, nordestinos, de baixa renda e alta dependência de programas sociais) apresentou menor probabilidade de consulta com dentista nos últimos 2 anos (OR 0,59; IC95% 0,48-0,73; p<0,001); Perfil B (meninos, brancos, nordestinos, de baixa renda e beneficiários de programas sociais) apresentou piores condições quanto à presença de água canalizada no domicílio (OR 0,38; IC95% 0,19-0,76; p=0,006); e Perfil C (meninas, negras, nortistas, com renda mais alta e menor dependência de programas sociais) concentrou as melhores condições de consulta e água canalizada (p<0,001).

**Conclusão:**

Cárie dentária não tratada em crianças de 5 anos reflete iniquidades estruturais que combinam desigualdades de raça, classe, território e renda.

## INTRODUÇÃO

A cárie dentária é uma das doenças crônicas mais prevalentes no mundo, gerando impactos clínicos, funcionais e psicossociais relevantes ao longo do curso da vida^1^. Apesar de passível de prevenção e tratamento, sua distribuição é marcada por importantes desigualdades sociais, econômicas e territoriais, refletindo padrões de iniquidade em saúde observados tanto em países de alta quanto de baixa renda^2^ ^,^ ^3^ ^,^ ^4^ ^,^ ^5^.

Em 2019, aproximadamente 514 milhões de crianças apresentavam cárie dentária não tratada na dentição decídua no mundo, correspondendo a uma prevalência média global de 43%, com valores superiores a 40% em mais de 2/3 dos países^3^. No contexto brasileiro, a cárie dentária persiste como um agravo prioritário na infância, particularmente aos 5 anos de idade, faixa etária utilizada internacionalmente como referência para monitoramento epidemiológico da cárie precoce e suas consequências^6^ ^,^ ^7^.

Evidências sugerem que a ocorrência e a persistência da cárie estão fortemente associadas aos determinantes sociais da saúde, que operam por meio de gradientes relacionados a renda, escolaridade, raça/cor, condições de vida, acesso à água potável e disponibilidade de serviços de saúde^2^ ^,^ ^4^ ^,^ ^8^ ^,^ ^9^. Esses determinantes não incidem de maneira isolada; ao contrário, tendem a se sobrepor e interagir entre si, produzindo padrões mais complexos de vulnerabilidade à medida que marcadores sociais como raça/cor, classe social e território se acumulam^3^ ^,^ ^5^ ^,^ ^8^.

Nesse sentido, a perspectiva da interseccionalidade, introduzida originalmente por Crenshaw e posteriormente expandida para o campo da saúde coletiva^8^, tem emergido como um referencial relevante para compreender como eixos de desigualdade se articulam na produção das iniquidades. No campo da saúde bucal, pesquisas recentes têm defendido a incorporação da interseccionalidade ao estudo das desigualdades, destacando que explicações baseadas em determinantes isolados subestimam a complexidade dos processos sociais envolvidos^8^ ^,^ ^10^.

No entanto, a aplicação da interseccionalidade na epidemiologia da saúde bucal ainda é limitada. A maior parte das investigações adota abordagens metodológicas que tratam os determinantes como variáveis isoladas, dificultando a captura de padrões combinados de desvantagem social^7^. Nesse cenário, técnicas de agrupamento, como o *Two-Step Cluster*, têm sido recomendadas como alternativa metodológica para identificar perfis de vulnerabilidade, permitindo analisar como marcadores sociais se combinam na produção das iniquidades^3^. Apesar de úteis, essas estratégias permanecem subexploradas na literatura odontológica, sobretudo na infância.

A realidade brasileira oferece um contexto relevante para tais análises. O país apresenta profundas desigualdades raciais, territoriais e socioeconômicas historicamente estruturadas, que repercutem no uso, na oportunidade e na resolutividade do cuidado em saúde bucal^6^ ^,^ ^9^. Estudos recentes sugerem que crianças negras, indígenas, pobres ou residentes em regiões menos desenvolvidas enfrentam barreiras acumuladas ao cuidado odontológico, mesmo no contexto do Sistema Único de Saúde (SUS)^4^.

Além disso, dados nacionais indicaram que a prevalência de crianças de 5 anos com pelo menos um dente decíduo com cárie dentária não tratada passou de 53,4% em 2010 para 41,2% em 2023, evidenciando redução no período, porém com persistência de importantes desigualdades sociais e territoriais^5^.

Nesse contexto, o Levantamento Nacional de Saúde Bucal (SB Brasil 2023) representa uma oportunidade para investigar desigualdades em saúde bucal na infância, dada sua abrangência nacional, metodologia padronizada e disponibilidade de variáveis sociais, clínicas e assistenciais^5^. Apesar disso, análises interseccionais baseadas nessa base de dados ainda não foram exploradas.

Diante das lacunas identificadas, o presente estudo tem como objetivos identificar perfis interseccionais de vulnerabilidade em crianças de 5 anos com cárie dentária não tratada e analisar suas desigualdades sociais.

## MÉTODOS

### Delineamento

Trata-se de um estudo transversal, de base populacional e caráter analítico, realizado com base nos dados do SB Brasil 2023.

### Participantes

A população do estudo foi composta por crianças de ambos os sexos, na idade de 5 anos, examinadas no inquérito (amostra nacional, representativa por Unidade da Federação (UF), região, capital/interior), sendo excluídos os registros com ausência de dados sobre a experiência de cárie dentária, inconsistências nos formulários ou falta de informação nas variáveis demográficas, socioeconômicas e territoriais essenciais.

### Variáveis

O índice ceo-d, que quantifica dentes decíduos cariados, extraídos por cárie ou obturados, foi utilizado como indicador de experiência de cárie. Crianças com ceo-d=0 foram classificadas como “sem dentes com cárie”. Para a classificação “≥1 dente com cárie”, utilizou-se apenas o componente “cariado” do índice, considerando-se a presença de pelo menos um dente decíduo com lesão ativa de cárie (c>0).

Para a identificação dos perfis interseccionais, foram considerados apenas os registros de crianças com cárie dentária não tratada (componente cariado >0). Foram selecionadas variáveis sociais e demográficas com base na literatura sobre determinação social da saúde e interseccionalidade, incluindo as variáveis categóricas raça/cor da pele (branca, negra^11^ [pretos e pardos], amarela e indígena); renda familiar mensal (menor que 1 salário mínimo, de 1 a 2 salários mínimos e maior que 2 salários mínimos); escolaridade da mãe (não estudou/Educação de Jovens e Adultos [EJA], ensino fundamental incompleto ou completo, ensino médio incompleto ou completo, ensino superior incompleto ou completo); região de residência (Norte, Nordeste, Centro-Oeste, Sudeste e Sul); e participação em programas sociais (sim ou não), incluindo o Programa Bolsa Família e o Benefício Assistencial de Prestação Continuada.

Após a formação dos perfis, procederam-se análises comparativas entre os grupos identificados utilizando variáveis relacionadas a condições de vida e acesso aos serviços, considerando-se a última consulta com dentista (nunca foi ao dentista, nos últimos 2 anos e há mais de 2 anos), empregada como proxy de acesso ao cuidado, e a presença de água canalizada no domicílio (com encanamento ou sem encanamento), utilizada como proxy de infraestrutura domiciliar.

As categorias seguiram a padronização do SB Brasil 2023 e foram mantidas na forma original ou agrupadas conforme relevância epidemiológica^12^ ^,^ ^13^.

### Fonte de dados

Os dados utilizados são oriundos do SB Brasil 2023. Os dados brutos do SB Brasil 2023 foram obtidos junto à Coordenação-Geral de Saúde Bucal do Ministério da Saúde, mediante solicitação formal. Essa base reúne informações demográficas, socioeconômicas, territoriais, clínicas e relativas ao uso de serviços odontológicos, coletadas de forma padronizada em todo o território nacional.

Para avaliação da qualidade dos dados e redução de potenciais vieses, foram realizadas verificações de completude e consistência lógica das informações. Registros referentes a crianças sem informação sobre o índice ceo-d ou com inconsistências nas respostas foram excluídos previamente às análises estatísticas.

### Amostra do estudo

Considerou-se a totalidade de registros disponíveis para o período, garantindo a representatividade da amostra. Essa abordagem evitou a amostragem aleatória, já que os dados eram secundários e abrangiam um universo significativo da população-alvo.

### Métodos estatísticos

Na análise descritiva, foram calculadas frequências absolutas e relativas das características demográficas, socioeconômicas, territoriais e clínico-assistenciais da amostra total. As associações bivariadas entre a presença de cárie e os marcadores sociais foram testadas na amostra total pelo teste de qui-quadrado de Pearson, adotando-se um nível de significância de 5%.

Para identificar perfis interseccionais de vulnerabilidade, realizou-se uma análise de agrupamento do tipo *Two-Step Cluster*, utilizando as variáveis sociais e demográficas previamente especificadas. A medida de distância *log-likelihood* foi empregada, e o Critério de Informação Bayesiano (BIC) orientou a seleção do número ótimo de *clusters*. Os perfis formados foram descritos segundo suas características predominantes.

Na etapa subsequente, investigaram-se diferenças entre os perfis quanto às condições de vida e acesso ao cuidado. Essas análises foram conduzidas exclusivamente entre os indivíduos classificados nos perfis, dado que a variável latente “perfil” não é atribuível aos casos incompletos e não pode ser imputada sem distorcer a estrutura probabilística dos agrupamentos.

Utilizou-se regressão logística binária para a variável “água canalizada no domicílio” (referência: sem encanamento) e a regressão logística multinomial para a variável “consulta odontológica” (referência: nunca foi ao dentista). As categorias de referência foram escolhidas por representarem as situações de maior vulnerabilidade e serem epidemiologicamente relevantes como linha de base comparativa.

Foram estimadas razões de chances (*Odds Ratio* - OR) e Intervalos de Confiança de 95% (IC95%). Os modelos foram estimados de forma não ajustada, uma vez que os perfis resultam da combinação simultânea dos marcadores sociais incorporados previamente na análise de *cluster*, não sendo necessária a inclusão desses mesmos marcadores como covariáveis adicionais nos modelos subsequentes.

Todas as análises estatísticas foram realizadas no software *IBM SPSS Statistics*, versão 23.0 (IBM Corp., Armonk, NY, Estados Unidos), e conduzidas sem aplicação de pesos amostrais, por se tratar de exploração interna de perfis sociais e não de estimativas populacionais.

O estudo seguiu as recomendações da *Strengthening the Reporting of Observational Studies in Epidemiology* (STROBE)^14^, que orienta a transparência e a completude na apresentação de estudos observacionais.

## Declaração de disponibilidade de dados

Todo o conjunto de dados que dá suporte aos resultados deste estudo está disponível mediante solicitação ao autor correspondente. O conjunto de dados não está publicamente disponível devido à integração de bancos de dados realizados pelos autores.

## RESULTADOS

Do total de 40.720 indivíduos registrados no SB Brasil 2023, 7.198 eram crianças de 5 anos, das quais 7.185 (17,6%) foram incluídas na análise após a exclusão de 13 registros sem informação sobre o índice ceo-d.

No perfil demográfico, socioeconômico e territorial (Tabela 1), observou-se distribuição equilibrada entre os sexos (51,1% meninos; 48,9% meninas) e predominância de residentes nas regiões Nordeste (36,6%) e Norte (24,6%). Quanto à raça/cor, 61,9% foram classificadas como negras (pretas e pardas) e 34,6%, como brancas. Em relação à escolaridade da mãe, 40,7% tinham ensino médio completo ou incompleto e 15,5%, ensino superior. Além disso, 48,7% das famílias eram beneficiárias de programas sociais. Do ponto de vista clínico-assistencial, 40,1% das crianças nunca haviam consultado um dentista, e 44,8% apresentavam cárie dentária não tratada.

**Tabela 1.** Características demográficas, socioeconômicas, territoriais e clínico-assistenciais de crianças de 5 anos. Brasil, 2023 (n=7.185).

Características	n (%)
Sexo	
Masculino	3.670 (51,1)
Feminino	3.515 (48,9)
Região	
Norte	1.767 (24,6)
Nordeste	2.633 (36,6)
Sudeste	842 (11,7)
Sul	859 (12,0)
Centro-Oeste	1084 (15,1)
Raça/cor da pele	
Branca	2.488 (34,6)
Negra	4.450 (61,9)
Amarela	76 (1,1)
Indígena	24 (0,3)
Não informado	147 (2,0)
Escolaridade da mãe	
Não estudou/Educação de Jovens e Adultos	658 (9,2)
Ensino fundamental incompleto ou completo	1.847 (25,7)
Ensino médio incompleto ou completo	2.926 (40,7)
Ensino superior incompleto ou completo	1.115 (15,5)
Não informado	639 (8,9)
Renda (salário mínimo)^a^	
Menor que 1	1.978 (27,5)
De 1 a 2	1.661 (23,1)
Maior que 2	1.158 (16,1)
Não informado	2.388 (33,2)
Família com participação em programa social	
Sim	3.499 (48,7)
Não	3.029 (42,2)
Não informado	657 (9,1)
Última consulta com dentista	
Nunca foi ao dentista	2.878 (40,1)
Nos últimos 2 anos	3.658 (50,9)
Há mais de 2 anos	334 (4,6)
Não informado	315 (4,4)
Água canalizada no domicílio	
Com encanamento	6.570 (91,4)
Sem encanamento	171 (2,4)
Não informado	444 (6,2)
Cárie	
Sem dentes com cárie	3.965 (55,2)
≥1 dente com cárie	3.220 (44,8)

*A variável de renda familiar foi autorreferida em faixas de salários mínimos. Durante o período de coleta do SB Brasil 2023 (março/2022 a março/2024), o salário mínimo brasileiro variou de R$1.212,00 (2022), R$1.302,00 (jan.-abr./2023) e R$1.320,00 (maio/2023-2024).

Na análise bivariada (Tabela 2), verificou-se associação significativa entre cárie dentária não tratada e sexo, região, raça/cor, escolaridade da mãe, renda familiar, participação em programas sociais e presença de água canalizada no domicílio (p<0,05).

**Tabela 2.** Associação entre as características demográficas, socioeconômicas, territoriais e presença de cárie dentária não tratada em crianças de 5 anos. Brasil, 2023 (n=7.185).

Características	Sem dentes com cárie n (%)	≥1 dente com cárie n (%)	p-valor
Sexo			
Masculino	1.982 (54,0)	1.688 (46)	0,040
Feminino	1.983 (56,4)	1.532 (43,6)	
Região			
Norte	773 (43,7)	994 (56,3)	<0,001
Nordeste	1.416 (53,8)	1.217 (46,2)	
Sudeste	576 (68,4)	266 (31,6)	
Sul	563 (65,5)	296 (34,5)	
Centro-Oeste	637 (58,8)	447 (41,2)	
Raça/cor da pele			
Branca	1.607 (64,6)	881 (35,4)	<0,001
Negra	2.229 (50,1)	2.221 (49,9)	
Amarela	43 (56,6)	33 (43,4)	
Indígena	6 (25,0)	18 (75,0)	
Escolaridade da mãe			
Não estudou/Educação de Jovens e Adultos	354 (53,8)	304 (46,2)	<0,001
Ensino fundamental incompleto ou completo	888 (48,1)	959 (51,9)	
Ensino médio incompleto ou completo	1.586 (54,2)	1.340 (45,8)	
Ensino superior incompleto ou completo	804 (72,1)	311 (27,9)	
Renda (salário mínimo)			
Menor que 1	908 (45,9)	1.070 (54,1)	<0,001
De 1 a 2	875 (52,7)	786 (47,3)	
Maior que 2	818 (70,6)	340 (29,4)	
Família com participação em programa social			
Sim	1.644 (47,0)	1.855 (53,0)	<0,001
Não	1.938 (64,0)	1.091 (36,0)	
Última consulta com dentista			
Nunca foi ao dentista	1.605 (55,8)	1.273 (44,2)	0,715
Nos últimos 2 anos	2.003 (54,8)	1.655 (45,2)	
Há mais de 2 anos	185 (55,4)	149 (44,6)	
Água canalizada no domicílio			
Com encanamento	3.610 (54,9)	2.960 (45,1)	<0,001
Sem encanamento	69 (40,4)	102 (59,6)	

A prevalência de cárie foi maior entre meninos (46%) do que entre meninas (43,6%); entre residentes das regiões Norte (56,3%) e Nordeste (46,2%), em comparação ao Sudeste (31,6%) e Sul (34,5%); entre crianças indígenas (75%) e negras (49,9%), em comparação às brancas (35,4%). A menor prevalência ocorreu entre filhos de mães com ensino superior (27,9%), enquanto a maior ocorreu entre filhos de mães com ensino fundamental (51,9%). A prevalência também foi maior em famílias com renda familiar menor que 1 salário mínimo (54,1%), em beneficiários de programas sociais (53%) e em domicílios sem água canalizada (59,6%) (p<0,001). A variável “última consulta odontológica” não apresentou associação estatisticamente significativa com cárie (p=0,715) (Tabela 2).

Na análise de *Two-Step Cluster* (Tabela 3), as variáveis com maior peso para definição dos perfis foram participação em programas sociais, raça/cor e renda familiar, enquanto a região, escolaridade da mãe e o sexo tiveram menor contribuição. Identificaram-se três perfis interseccionais: Perfil A, composto majoritariamente por meninos (55,7%), residentes no Nordeste (49,4%), negros (100%), filhos de mães com ensino médio incompleto ou completo (45,3%), com renda familiar menor que 1 salário mínimo (66%) e participação em programas sociais (95,9%); Perfil B, composto principalmente por meninos (50,2%), residentes no Nordeste (41,5%), brancos (92%), filhos de mães com ensino médio incompleto ou completo (52,3%), com renda familiar menor que 1 salário mínimo (63,1%) e participação em programas sociais (90,8%); e Perfil C, composto principalmente por meninas (51,2%), residentes no Norte (31,4%), negras (64,4%), filhas de mães com ensino médio incompleto ou completo (45%), com renda familiar maior que 2 salários mínimos (43,8%) e sem participação em programas sociais (91,3%).

**Tabela 3.** Frequência das características demográficas, socioeconômicas e territoriais, segundo os perfis de crianças de 5 anos com cárie não tratada. Brasil, 2023 (n=3.220).

Características	Perfil A 50,6% (1.030)	Perfil B 16,0% (325)	Perfil C 33,5% (682)
Sexo			
Masculino	55,7	50,2	48,8
Feminino	44,3	49,8	51,2
Região			
Norte	33,5	22,8	31,4
Nordeste	49,4	41,5	20,4
Sudeste	3,6	4,9	11,6
Sul	2,7	17,5	17,9
Centro-Oeste	10,8	13,2	18,8
Raça/cor da pele			
Branca	0,0	92,0	34,8
Negra	100	0,0	64,4
Amarela	0,0	4,9	0,3
Indígena	0,0	3,1	0,6
Escolaridade da mãe			
Não estudou/Educação de Jovens e Adultos	8,4	8,0	11,1
Ensino fundamental incompleto ou completo	41,7	35,7	21,7
Ensino médio incompleto ou completo	45,3	52,3	45,0
Ensino superior incompleto ou completo	4,5	4,0	22,1
Renda (salário mínimo)			
Menor que 1	66,0	63,1	14,8
De 1 a 2	34,0	30,8	41,3
Maior que 2	0,0	6,2	43,8
Família com participação em programa social			
Sim	95,9	90,8	8,7
Não	4,1	9,2	91,3

Na comparação entre perfis e indicadores de acesso e condições de vida (Tabela 4), crianças do Perfil C apresentaram maior proporção de consultas odontológicas recentes, enquanto os Perfis A e B concentraram as menores frequências de utilização de serviços. Em relação à infraestrutura domiciliar, o Perfil B apresentou maior proporção de domicílios sem água canalizada.

**Tabela 4.** Comparação dos perfis de crianças de 5 anos com cárie dentária não tratada, segundo última consulta com dentista e a presença de água canalizada no domicílio. Brasil, 2023 (n=3.220).

Perfil	Última consulta com dentista				Água canalizada no domicílio		
	Nunca foi ao dentista (%)	Nos últimos 2 anos (%)	Há mais de 2 anos (%)	p-valor^§^	Com encanamento (%)	Sem encanamento (%)	p-valor^§^
Perfil A*	47,7	48,0	4,4	< 0,001	96,8	3,2	0,018
Perfil B^†^	43,2	51,2	5,6		94,4	5,6	
Perfil C^‡^	35,0	59,2	5,8		97,8	2,2	

*sexo masculino, do Nordeste, negros, mãe com ensino médio incompleto ou completo, renda familiar menor que um salário mínimo e com participação em programa social; ^†^sexo masculino, do Nordeste, brancos, mãe com ensino médio incompleto ou completo, renda familiar menor que um salário mínimo e participação em programa social; ^‡^sexo feminino, do Norte, negras, mãe com ensino médio incompleto ou completo, renda familiar maior que dois salários mínimos e sem participação em programa social; ^§^χ^2^ de Pearson.

As análises de regressão logística (Tabela 5) mostraram que os Perfis A e B apresentaram menores chances de acesso tanto a serviços odontológicos quanto a água canalizada, em comparação ao Perfil C. A diferença foi mais pronunciada no Perfil A para acesso odontológico e no Perfil B para acesso à água canalizada.

**Tabela 5.** Razão de chances e intervalos de confiança de 95% da última consulta com dentista e da presença de água canalizada no domicílio, segundo os perfis de crianças de 5 anos com cárie dentária não tratada. Brasil, 2023 (n=3.220).

Perfil	Categoria	OR	IC95%	p-valor^§^
Água canalizada no domicílio				
Perfil A*	Sem encanamento	0,68	0,37-1,26	0,217
Perfil B^†^	Sem encanamento	0,38	0,19-0,76	0,006
Perfil C^‡^	Ref.	-	-	-
Última consulta com dentista				
Perfil A*	Há mais de 2 anos	0,55	0,35-0,87	0,011
Perfil B^†^	Há mais de 2 anos	0,78	0,43-1,42	0,415
Perfil C^‡^	Ref.	-	-	-
Perfil A*	Nos últimos 2 anos	0,59	0,48-0,73	<0,001
Perfil B^†^	Nos últimos 2 anos	0,70	0,53-0,93	0,012
Perfil C^‡^	Ref.	-	-	-

OR: odds ratio; IC: intervalo de confiança.

*sexo masculino, do Nordeste, negros, mãe com ensino médio incompleto ou completo, renda familiar menor que um salário mínimo e com participação em programa social; ^†^sexo masculino, do Nordeste, brancos, mãe com ensino médio incompleto ou completo, renda familiar menor que um salário mínimo e participação em programa social; ^‡^sexo feminino, do Norte, negras, mãe com ensino médio incompleto ou completo, renda familiar maior que dois salários mínimos e sem participação em programa social; ^§^para última consulta com dentista, regressão logística multinomial (categoria de referência: nunca foi ao dentista). Para água canalizada no domicílio, regressão logística binária (categoria de referência: sem encanamento).

## DISCUSSÃO

Este estudo encontrou alta prevalência de cárie dentária não tratada em crianças de 5 anos no Brasil, com maiores frequências entre crianças negras e indígenas, residentes nas regiões Norte e Nordeste, de baixa renda e beneficiárias de programas sociais. A análise interseccional identificou três perfis distintos de vulnerabilidade, sendo o Perfil A o mais vulnerável e o Perfil C, o menos vulnerável. Observou-se menor probabilidade de consulta odontológica e pior acesso à água canalizada entre os perfis mais vulneráveis.

A distribuição desigual da cárie dentária não tratada no país indica que esse agravo reflete padrões estruturais de desigualdade, conforme já apontado por investigações nacionais e internacionais que associam o agravo a condições de vulnerabilidade social^4^ ^,^ ^10^ ^,^ ^15^ ^,^ ^16^ ^,^ ^17^. A alta prevalência observada, especialmente nas regiões Norte e Nordeste e entre crianças negras e indígenas, demonstrou que a não resolução do agravo reflete a persistência das desigualdades estruturais na oferta e no acesso ao cuidado odontológico^18^ ^,^ ^19^ ^,^ ^20^. Esses resultados sugerem que a cárie dentária não tratada, mais do que um problema clínico, é uma expressão concreta da determinação social da saúde^21^ ^,^ ^22^ ^,^ ^23^ ^,^ ^24^.

A análise interseccional dos dados, operacionalizada pela identificação de três perfis de vulnerabilidade, reforça essa compreensão. O Perfil A, composto predominantemente por crianças negras, de baixa renda, baixa escolaridade da mãe, majoritariamente residentes no Nordeste e com alta dependência de programas sociais, representa o ponto de grande vulnerabilidade social^25^ ^,^ ^26^. A sobreposição de marcadores raciais, econômicos e territoriais configura maior vulnerabilidade ao não tratamento da cárie dentária, o que é compatível com estudos que apontam barreiras estruturais no acesso e na continuidade do cuidado de populações historicamente marginalizadas^6^ ^,^ ^9^ ^,^ ^18^ ^,^ ^19^ ^,^ ^20^.

O Perfil B, formado majoritariamente por crianças brancas, apresentou vulnerabilidade socioeconômica e maior ausência de água canalizada, indicando que a pobreza configura barreiras estruturais à saúde bucal^27^. Contudo, difere do Perfil A, em que a vulnerabilidade econômica se soma a marcadores raciais e territoriais, ampliando desigualdades^8^ ^,^ ^10^. No contexto brasileiro, evidências mostram que a pobreza não é vivenciada de forma homogênea entre grupos raciais, refletindo efeitos interseccionais de cor/raça e classe^28^.

O Perfil C, ainda que com melhores condições econômicas e de acesso, não está isento das marcas da desigualdade racial, o que demonstra que a raça segue sendo um eixo estruturante das oportunidades de cuidado, conforme discutido em estudos que aplicam o conceito de interseccionalidade à saúde bucal^10^ ^,^ ^25^ ^,^ ^29^ ^,^ ^30^.

A cárie dentária não tratada surge, portanto, como resultado do acúmulo de desvantagens sociais. A associação direta com baixa renda, menor escolaridade da mãe e recebimento de benefício social reafirma que o tratamento odontológico, mesmo no contexto do SUS, não se realiza de modo universal nem equitativo^27^ ^,^ ^31^ ^,^ ^32^. As famílias em situação de pobreza têm menos condições de acessar serviços preventivos e reabilitadores, além de enfrentarem barreiras logísticas, culturais e institucionais^33^ ^,^ ^34^.

A baixa escolaridade familiar limita a capacidade de compreender a importância da atenção precoce, enquanto a vulnerabilidade econômica restringe o tempo e os meios para buscar atendimento^35^ ^,^ ^36^. Embora a escolaridade da mãe tenha sido associada à cárie dentária não tratada no presente estudo, chama a atenção a distribuição relativamente homogênea dessa variável na amostra, com predominância de ensino médio completo ou incompleto e presença de um contingente com ensino superior. Essa composição diminui o contraste educacional entre os subgrupos, o que pode ter reduzido seu poder discriminatório na formação dos perfis. Esse achado é consistente com mudanças nas últimas décadas relacionadas à expansão da escolarização feminina no Brasil, nas quais maiores níveis formais de escolaridade podem coexistir com vulnerabilidade econômica e racial^37^ ^,^ ^38^.

A relação entre infraestrutura domiciliar e saúde bucal merece destaque. A ausência de água canalizada esteve associada a maior prevalência de cárie dentária não tratada, indicando que condições materiais de vida integram o conjunto de fatores que moldam a possibilidade de higiene bucal adequada e uso de insumos como dentifrício fluoretado^5^ ^,^ ^16^. Nesse sentido, o agravo observado não pode ser interpretado apenas em termos clínicos, mas como expressão de desigualdades estruturais de acesso a direitos básicos, especialmente relevantes na primeira infância^21^ ^,^ ^26^.

Outro achado relevante foi a ausência de associação entre ter consultado o dentista e não apresentar cárie dentária não tratada. Esse resultado sugere que a atenção à saúde bucal na infância não depende exclusivamente do contato pontual com o serviço odontológico, mas de fatores que envolvem continuidade do cuidado, organização da rede de atenção, educação em saúde, apoio familiar e condições de vida. A consulta isolada não assegura resolutividade do cuidado, o que é compatível com análises que apontam limitações estruturais e organizacionais na atenção básica, demanda reprimida e práticas centradas em urgência e procedimentos mutiladores^18^ ^,^ ^19^ ^,^ ^27^ ^,^ ^28^ ^,^ ^31^ ^,^ ^39^.

A cárie dentária não tratada pode ser compreendida, portanto, como expressão das iniquidades no cuidado, e não apenas como falha de prevenção. Enquanto o acesso inicial aos serviços pode ter se expandido, a capacidade de resolutividade e continuidade ainda é desigual^29^. O não tratamento do agravo representa, assim, o estágio final da exclusão: adoecer e não ser tratado. Esse fenômeno revela uma desigualdade qualitativa, uma “iniquidade na resolutividade”, na qual grupos vulneráveis têm menor chance de concluir o tratamento e alcançar reabilitação.

O fato de as variáveis raça/cor, escolaridade da mãe, renda e programa social terem maior peso na formação dos perfis de cárie dentária não tratada do que fatores clínicos ou assistenciais reforça o entendimento sobre a determinação social da saúde bucal^5^ ^,^ ^22^ ^,^ ^35^ ^,^ ^36^. A interseccionalidade contribui para compreender que esses processos não atuam isoladamente - o efeito da pobreza é agravado pelo racismo, e ambos são intensificados por contextos territoriais de exclusão. Essa abordagem rompe com leituras tradicionais que isolam variáveis, mostrando que os determinantes sociais se interagem mutuamente^30^ ^,^ ^32^.

A infância é um período em que as desigualdades podem se manifestar de forma precoce. A presença de cárie dentária não tratada aos 5 anos não representa apenas um problema clínico imediato, mas um indicador de acúmulo precoce de vulnerabilidades que podem afetar o desenvolvimento escolar, a autoestima, a nutrição e o bem-estar geral^24^ ^,^ ^40^. A persistência dessas desigualdades desde a primeira infância aponta para um processo de injustiça geracional, no qual as condições sociais adversas na infância moldam trajetórias de vida mais desiguais^35^.

Este estudo apresenta limitações inerentes ao uso de dados secundários do SB Brasil 2023^5^, como a dependência de informações padronizadas e a ausência de variáveis contextuais mais amplas, por exemplo, cobertura de equipes de saúde bucal, características da atenção primária e indicadores ambientais locais^21^ ^,^ ^35^. Além disso, a variável “última consulta com dentista” não permite distinguir o tipo de atendimento (preventivo, curativo ou emergencial), o que pode subestimar desigualdades qualitativas no cuidado^18^.

Outro aspecto relevante refere-se à estrutura das variáveis analisadas, que apresentam níveis distintos (familiar, domiciliar e territorial) e foram modeladas no mesmo nível individual, o que pode gerar perda de informação contextual. Somado a isso, a natureza transversal do estudo impossibilita estabelecer relações causais entre determinantes sociais e a experiência de cárie dentária não tratada, devendo os achados ser interpretados com cautela.

Como ponto forte, destaca-se o uso de base de abrangência nacional, metodologia padronizada e aplicação de análise interseccional por meio da análise de *Two-Step Cluster*, que operacionalizou empiricamente dimensões sociais e raciais no campo da saúde bucal^25^ ^,^ ^29^ ^,^ ^30^. Essa estratégia permitiu capturar o entrelaçamento de dimensões sociais e raciais, oferecendo uma leitura mais densa e contextualizada das iniquidades. Estudos futuros podem avançar ao incorporar variáveis contextuais, como densidade de equipes de saúde bucal, cobertura da Estratégia Saúde da Família (ESF) e indicadores de infraestrutura urbana, para compreender níveis mais amplos de determinação social^21^ ^,^ ^27^.

Os presentes achados possuem implicações diretas para as políticas públicas. O enfrentamento da cárie dentária não tratada exige estratégias que combinem ações clínicas com intervenções estruturais^36^ ^,^ ^41^. É necessário fortalecer a atenção primária em saúde bucal, ampliar a fluoretação das águas, desenvolver ações educativas antirracistas e interseccionais e garantir o acompanhamento longitudinal das crianças mais vulneráveis^22^ ^,^ ^23^ ^,^ ^42^ ^,^ ^43^. As políticas devem reconhecer explicitamente os marcadores sociais de desigualdade, incorporando a equidade racial, territorial e social como critérios de priorização.

A cárie dentária não tratada configura-se, portanto, como um espelho das desigualdades brasileiras, concentrando-se entre crianças negras, pobres e residentes nas regiões Norte e Nordeste, o que aponta para uma distribuição desigual do direito à saúde e à infância^4^ ^,^ ^16^ ^,^ ^26^ ^,^ ^32^. A análise de perfis mostrou que a intersecção de marcadores sociais - como raça/cor, renda, escolaridade materna e território - não é aditiva, mas produz arranjos específicos de vulnerabilidade, reafirmando que o agravo expressa a determinação social da saúde. Esses resultados indicam que seu enfrentamento demanda políticas públicas orientadas à equidade racial, territorial e social e um SUS capaz de reduzir desigualdades desde os primeiros anos de vida.
